# Association between Oral Health Status and Dementia

**Published:** 2019-05

**Authors:** Eun-Sol KIM, Eun-Deok JO, Gyeong-Soon HAN

**Affiliations:** 1. Department of Health Science, Graduate School, Gachon University, Incheon, Republic of Korea; 2. Department and Research Institute of Dental Biomaterials and Bioengineering, College of Dentistry, Yonsei University, Seoul, Republic of Korea

## Dear Editor-in-Chief

As the prevalence rate of dementia in the elderly increases, to facilitate the early detection and management of dementia, it is important to detect cognitive impairment early by using simple and appropriate tools and to identify and manage related factors. Among oral diseases, it is associated with dementia and periodontal disease and is caused by biofilm (1). Therefore, the purpose of this study was to evaluate the cognitive abilities, socio-demographic characteristics, and various oral health-related indices in the elderly population vulnerable to dementia, to understand the influence of cognitive abilities on oral health, and to utilize this information it for health promotion among the elderly.

This study was approved by the IRB of Gachon University (1044396-201704-HR-077-01).

Subjects were 394 elderly people from May 9 to Jun 23, 2017. The researcher asked the subject directly to the questionnaire and wrote it when the subject answered. The questionnaire consisted of socio-demographic characteristics, cognitive status, and subjective oral status. Cognitive status was assessed using the translated version (2) of the Mini-Mental State Examination. Oral examinations were performed to confirm the dental plaque index and the numbers of remaining and decayed teeth of the subjects.

The collected data were analyzed using SPSS WIN 19.0 (IBM Co., Armonk, NY, USA). The Chi-square test and a binary logistic regression analysis was conducted to examine the data. Table 1 presents that dementia screening scores is a significant difference in gender, age, educational level, and residence status. In oral health status, there is a significant difference with remaining tooth, dental plaque index, halitosis self-awareness, and food impaction. The results of the binary logistic regression analysis with cognitive function as the dependent variable have been shown in Table 2. The results of the binary logistic regression analysis with cognitive function as the dependent variable have been shown in Table 2. Group aged ≥ 80 yr was 3.177 times more likely to have cognitive impairment as compared to the < 80 yr group. The educated group was 89% less likely to have cognitive impairment than the uneducated group, and the probability of occurrence of cognitive impairment for those who lived with their family was 46.7% lower than that for those who lived alone. Age and education have increased the risk of cognitive impairment (3). In relation to residence status, the emotional and cognitive functions of the elderly living alone may not be adequately stimulated. Regression analysis showed that the cognitive impairment rates were 68% lower in the self-awareness halitosis group, this result is thought that person with low cognitive ability does not aware of halitosis.

**Table 1:** Cognitive impairment according to socio-demographic characteristics and oral health status

***Variables***		***N***	***MMSE<24 points n(%)***	***P***
Gender	Male	100	10 (10.0)	**<0.001**
	Female	294	120 (40.8)	
Age (yr)	< 75	136	16 (11.8)	**<0.001**
	75–84	178	62 (34.8)	
	≥ 85	80	52 (65.0)	
Education (yr)	0–3	154	102 (66.2)	**<0.001**
	≥ 4	240	28 (20.7)	
Residence status	Alone	212	88 (41.5)	**<0.001**
	With family	182	42 (23.1)	
General disease	Present	302	100 (33.1)	0.928
	Absent	92	30 (32.6)	
Remaining tooth (n)	0–10	106	50 (47.2)	**<0.001**
	11–20	96	34 (35.4)	
	≥ 21	192	46 (24.0)	
Decayed tooth (n)	Present	124	42 (33.0)	0.802
	Absence	270	88 (32.6)	
Dental plaque index	≤ 50%	112	23 (23.0)	**<0.001**
	>50%	233	83 (70.9)	
Bleeding	Present	362	124 (34.3)	0.074
	Absent	32	6 (18.8)	
Halitosis self-awareness	Present	294	108 (36.7)	**0.007**
	Absent	100	22 (22.0)	
Food impaction	Present	186	74 (39.8)	**0.007**
	Absent	208	56 (26.9)	
Total		394	130 (33.0)	

*P*-values obtained from the Chi-square test.

**Table 2:** Logistic regression for cognitive function by characteristics of subjects

***Independent variables***		***OR (95% CI)***	
Gender	(Male=0, Female=1)	1.294	(0.558–3.001)
Age (yr)	(<80=0, ≥80 =1)	3.177	(1.824–5.534)
Education	(Uneducated=0, Educated=1)	0.110	(0.036–0.334)
Living arrangement	(Alone=0, With Family=1)	0.533	(0.300–0.947)
General disease	(Absent=0, Present=1)	0.620	(0.312–1.230)
Remaining tooth (n)	(<17=0, ≥17=1)	0.943	(0.510–1.745)
Decayed tooth	(Absent=0, Present=1)	1.488	(0.845–2.623)
Dental plaque index	(≤50%=0, >50%=1)	2.304	(1.212–4.380)
Bleeding of gingiva	(Absent=0, Present=1)	0.610	(0.218–1.712)
Halitosis self-awareness	(Absent=0, Present=1)	0.320	(0.162–0.634)
Food impaction	(Absent=0, Present=1)	0.733	(0.402–1.338)

OR: Odds Ratio, CI: Confidence Interval

Model Chi-square=12.81, df =8, pseudo R-square=35.2% (Nagelkerke), *P*<0.001

Because the dental plaque is removed only by physical methods such as brushing, dental plaque management is likely to be insufficient as the level of cognitive impairment increases. In this study, cognitive impairment was 2.304 times more likely to occur in those with a dental plaque index of >50% as compared to that in those with an index of ≤50%. The lower the cognitive abilities were, the lower was the ability to maintain oral hygiene, and therefore, the higher was the risk of periodontal disease (4). We could confirm the relationship between dementia and oral health status. The providing of good oral health program to the elderly is beyond simply managing the oral health, therefore, the dementia-related program provided to the elderly should include the oral health program.

## References

[1] Okamoto N Morikawa M Okamoto K (2010). Tooth loss is associated with mild memory impairment in the elderly: the Fujiwara-kyo study. Brain Res, 1349: 68–75. 20599812 10.1016/j.brainres.2010.06.054

[2] Lee CS Shin SC (1993). Standardization of the mini-mental state examination (MMSE) in Korea. J Korean Neuropsychiatr Assoc, 32(6): 950–961.

[3] Ribeiro GR Costa JL Ambrosano GM (2012). Oral health of the elderly with Alzheimer’s disease. Oral Surg Oral Med Oral Pathol Oral Radiol, 114(3): 338–343. 22862974 10.1016/j.oooo.2012.03.028

[4] Lexomboon D Trulsson M Wrdh I (2012). Chewing Ability and Tooth Loss: Association with Cognitive Impairment in an Elderly Population Study. J Am Geriatr Soc, 60(10): 1951–1956. 23035667 10.1111/j.1532-5415.2012.04154.x

